# Effect of resveratrol supplementation on hepatic steatosis and cardiovascular indices in overweight subjects with type 2 diabetes: a double-blind, randomized controlled trial

**DOI:** 10.1186/s12872-022-02637-2

**Published:** 2022-05-10

**Authors:** Abbas Ali Sangouni, Shima Abdollahi, Hassan Mozaffari-Khosravi

**Affiliations:** 1 Department of Nutrition, School of Public Health, Shahid Sadoughi University of Medical Sciences, Yazd, Iran; 2 Nutrition and Food Security Research Center, Shahid Sadoughi University of Medical Sciences, Yazd, Iran; 3 Department of Nutrition, School of Public Health, North Khorasan University of Medical Sciences, Bojnurd, Iran; 4 Yazd Diabetic Research Center, Shahid Sadoughi University of Medical Sciences, Yazd, Iran

**Keywords:** Type 2 diabetes mellitus, Resveratrol, Steatosis, Cardiovascular risk

## Abstract

**Background:**

Patients with type 2 diabetes mellitus (T2DM) are prone to develop non-alcoholic fatty liver disease (NAFLD) and cardiovascular diseases (CVD). We aimed to investigate whether the resveratrol supplementation improves novel hepatic and cardiovascular indices in these patients.

**Methods:**

We conducted a double-blind, randomized controlled trial for 8 weeks. Seventy-six patients with T2DM were randomly assigned to receive 1000 mg/day resveratrol or placebo. Levels of lipid accumulation product (LAP), visceral adiposity index (VAI), Castelli risk index I (CRI-I), CRI-II and atherogenic coefficient (AC) were measured at the beginning and after intervention.

**Results:**

A total of 71 participants completed the trial. After adjusting for confounding factors including medications, diabetes duration, energy intake and physical activity, no significant difference was found between the intervention group and the control group in LAP (mean change: − 2.46 ± 23.3 vs. 1.43 ± 14.3; P = 0.43), VAI (mean change: − 0.25 ± 1.1 vs. − 0.02 ± 0.6; P = 0.47), CRI-I (mean change: − 0.25 ± 0.9 vs. − 0.09 ± 0.5; P = 0.79), CRI-II (mean change: − 0.23 ± 0.7 vs. − 0.06 ± 0.6; P = 0.38) and AC (mean change: − 0.25 ± 0.9 vs. − 0.09 ± 0.5; P = 0.79).

**Conclusions:**

Resveratrol supplementation had no effect on hepatic steatosis and cardiovascular indices. Further clinical trials, especially among subjects with dyslipidemia are needed to reach a firm conclusion. In addition, taking all medications should be controlled in future studies.

*Trial registration* The protocol was registered on 29/12/2017 at the Iranian clinical trials website (IRCT20171118037528N1) with URL: https://en.irct.ir/trial/27734.

## Background

The prevalence of type 2 diabetes mellitus (T2DM) is increasing, and has become a major public health problem [1]. In 2020, the worldwide prevalence of T2DM was estimated about 6.3% among adult population [2]. The main characteristics of T2DM are elevated blood glucose and decreased insulin sensitivity [3]. Non-alcoholic fatty liver disease (NAFLD) is strongly linked to T2DM, and is more prevalent in these patients [4, 5]. Insulin resistance, inflammation, dyslipidemia, oxidative stress and obesity are involved in the pathogenesis of both T2DM and NAFLD [6–8]. Recently, new non-invasive indices including lipid accumulation product (LAP) and visceral adiposity index (VAI) have been introduced [9, 10] that can accurately assess hepatic steatosis [9–13]. It is also well established that prevalence of CVD is high among patients with T2DM, and it is known as a main cause of death among patients with T2DM [14]. Castelli risk index (CRI) and atherogenic coefficient (AC) are developed based on lipid profile to estimate cardiovascular risk [15, 16].

Resveratrol (3,5,4′-trihydroxy-trans-stilbene), as a polyphenolic compound with antioxidant and anti-inflammatory properties, is mainly found in plants such as berries, red grapes, rhubarb and peanuts [17]. Recent studies have revealed the beneficial effect of resveratrol on glycemic status, insulin sensitivity, oxidative stress and inflammation [18–22]. In addition, experimental studies have suggested that resveratrol can improve the severity of NAFLD through attenuating obesity and dyslipidemia [23–27].

There are some clinical trials that evaluated the effect of resveratrol supplementation on cardiovascular risk factors [18, 28–30]; but, their findings are inconsistent. On the other hand, the results of studies that investigated the effect of resveratrol on severity of NAFLD are not integrated [22, 31–33]. In addition, there is no study evaluating the effect of resveratrol supplementation on hepatic steatosis in patients with T2DM. Accordingly, we designed a randomized controlled trial (RCT) to investigate the effect of resveratrol supplementation (1000 mg/d) on hepatic steatosis indices (LAP and VAI) and cardiovascular indices (CRI-I, CRI-II and AC) in patients with T2DM.

## Methods

### Sample size

The present article is a part of our study [34] that estimated the optimal sample size (equal to or more than 36 participants in each group) based on the peroxisome proliferator-activated receptor alpha (PPARα) gene expression in the peripheral blood mononuclear cells (PBMCs) [35], using a proposed formula for parallel clinical trials [36] by considering α = 0.05, a power of 80%, and assuming a 20% of drop-out rate. However, a retrospective power analysis was performed for outcomes of this article to assure the sample size is adequate to detect statistical significance, and adequate power was observed for all the interested outcomes except for CRI-I levels (power = 44% for CRI-I).

### Recruitment and eligibility screening

Endocrinologist-diagnosed patients with T2DM were identified and screened at the Diabetes Research Center affiliated with Shahid Sadoughi University of Medical sciences in Yazd, Iran. The inclusion criteria were aged 30–60 years, body mass index (BMI) ranging 25–30 kg/m^2^ and glycated hemoglobin (HbA1c) lower than 8%. The exclusion criteria were as follows: all types of cancer, kidney or liver failure, gastrointestinal ulcers, mental disorders, cardiovascular diseases, insulin therapy, pregnancy or lactation, consuming antioxidant supplements, fibrate lipid‐lowering agents, platelet aggregation inhibitor or anti-inflammatory medications, and also red wine consumption (for at least 6 months before the enrollment). Participants with compliance rate lower than 80%, and who were unwilling to continue the trial were dropped out from the study.

### Trial design

A double-blind, placebo-controlled, single-center, randomized clinical trial with two parallel study arms (the resveratrol and placebo groups) was conducted for 8 weeks. The study was in complete agreement with the Helsinki declaration, and the protocol approved by the medical ethics committee of Shahid Sadoughi University of Medical Sciences and Health Services, Yazd (IR.SSU.SPH.REC.1396.120). All the participants were fully aware about the study process and they were asked to signed the consent form. The protocol of trial was registered on 29/12/2017 at the Iranian clinical trials website under code number IRCT20171118037528N1, with URL: https://en.irct.ir/trial/27734.

Participants were enrolled using a stratified randomization process based on gender (male/female) and age (30–45 and 45–60 years), utilizing a random allocation software (random numbers table) [37]. Random allocation of the participants to the resveratrol and placebo groups was performed by a third person. The participants and investigators were all blinded to the intervention assignment until the end of the intervention. The intervention group received 1000 mg/d resveratrol (two capsules per day, each capsule provided 500 mg of 99.71% micronized trans‐resveratrol with particle size lower than 1.9 μm and without any inactive ingredients, fillers, flavoring agents, and additives (Mega‐Resveratrol, Danbury, USA)), and the control group received the same amount and appearance of capsules containing methylcellulose. Packaging and labeling the containers as A or B was performed by a third person. The number of capsules that were not consumed by the participants was recorded at the end of the trial, and the compliance rate of each participant was evaluated.

### Dietary intake and physical activity assessments

The participants were asked to record their foods and beverages intakes at baseline and end of the trial (two weekdays and one weekend day). The Nutritionist IV software (The Hearst Corporation, San Bruno, CA) was used to analyze the collected data [38].

Moreover, using a validated questionnaire [39], the physical activity was measured at baseline and end of the trial. This questionnaire provides metabolic equivalents (METs) based on the type and intensity of activities in nine different levels from sleep/rest (0.9 METs) to high-intensity activities (> 6 METs). Calculating METs/h was performed by multiplying the time spent on each activity by the MET value, and finally, MET/h per day obtained by adding the MET/h of each activity together.

### Anthropometric assessments

Anthropometric variables including height, weight, and waist circumference (WC) were measured using standard methods, before and after the study. Height of participants was assessed via a stadiometer (Seca, Hamburg, Germany) with an accuracy of 0.5 cm. Utilizing a bioelectrical impedance analyzer (Tanita BC‐418, Tokyo, Japan), weight was measured with light clothes and without shoes. Measuring WC was performed using a flexible tape with an accuracy of 0.5 cm. BMI was calculated using the following formula: weight (kg)/height squared (m^2^).

### Laboratory evaluations

An overnight fasting venous blood sample (10 mL) was obtained from all participants. Samples were centrifuged at a speed 3000 rpm, for 10 min at 25 °C (Eppendorf AG, Hamburg), and serums were immediately frozen at −70 °C. Total cholesterol (TC), triglycerides (TG), low density lipoprotein-cholesterol (LDL-c) and high density lipoprotein-cholesterol (HDL-c) concentrations were measured by an autoanalyzer (AVIDA 1800 chemistry system; Siemens, United Kingdom) and utilizing Pars Azmoon kits (Tehran, Iran), at baseline and end of the study.

### Hepatic steatosis and cardiovascular indices

The LAP [9], VAI [10], CRI-I [16], CRI-II [16] and AC [15] indices were calculated at the baseline and end of the study based on the following equations:

$${\text{LAP}}_{{{\text{men}}}} = \, \left( {{\text{WC }} - { 65}} \right) \, \times {\text{ TG}}.$$

$${\text{LAP}}_{{{\text{women}}}} = \, \left( {{\text{WC }} - { 58}} \right) \, \times {\text{ TG}}.$$

$${\text{VAI}}_{{{\text{men}}}} = \, \left[ {{\text{WC}}/{39}.{68 } + \, \left( {{1}.{88 } \times {\text{ BMI}}} \right)} \right] \, \times \, \left( {{\text{TG}}/{1}.0{3}} \right) \, \times \, \left( {{1}.{52}/{\text{HDL}} - {\text{c}}} \right).$$

$${\text{VAI}}_{{{\text{women}}}} = \, \left[ {{\text{WC}}/{36}.{58 } + \, \left( {{1}.{89 } \times {\text{ BMI}}} \right)} \right] \, \times \, \left( {{\text{TG}}/0.{81}} \right) \, \times \, \left( {{1}.{31}/{\text{HDL}} - {\text{c}}} \right).$$

$${\text{CRI}} - {\text{I }} = {\text{ TC}}/{\text{HDL}} - {\text{c}}.$$

$${\text{CRI}} - {\text{II }} = {\text{ LDL}} - {\text{c}}/{\text{HDL}} - {\text{c}}.$$

$${\text{AC }} = \, \left( {{\text{TC}} - {\text{HDL}} - {\text{c}}} \right)/{\text{HDL}} - {\text{c}}.$$

### Statistical analysis

We used the statistical package for social science (SPSS) software (Chicago, Illinois, USA) version 24 to perform statistical analyses. Comparing the qualitative variables between two groups was performed using chi-square test. We used an independent t-test to compare the means of variables between the two groups. Within group comparisons were performed utilizing paired t-test. Analysis of covariance (ANCOVA) was carried out to adjust the effect of confounding factors (medications, diabetes duration, energy intake and physical activity). P < 0.05 was considered significant. Further details of the study protocol have been previously reported [34].

## Results

### Characteristics of the participants

**Fig. 1:**
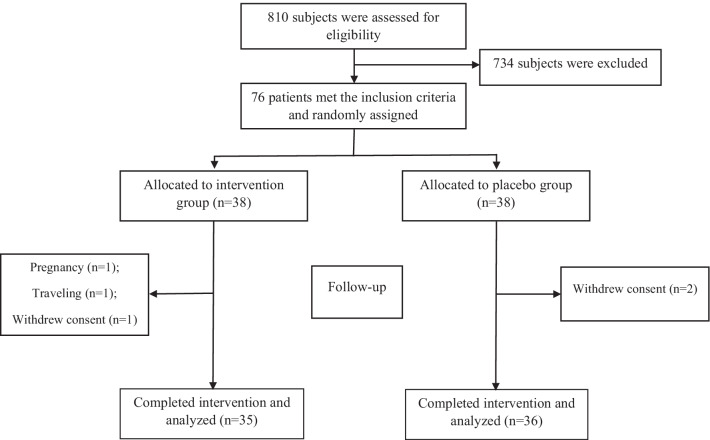
Flowchart of eligibility, screening and follow-up

A total of seventy-six patients were enrolled and randomly assigned into the intervention and control groups. Five patients left the trial due to the pregnancy (n = 1), traveling (n = 1), and withdrawal of consent (n = 3). Finally, 71 participants including 35 patients in the intervention group and 36 patients in the control group completed the trial (Fig. 1). All variables had normal distribution, and parametric tests were used to analyze data. The participants did not report any serious adverse event during intervention.

There were no significant differences between the intervention and control groups for the baseline measures, except for VAI which was significantly higher in the intervention group, compared to the control group (P = 0.04) (Table 1). In addition, no significant difference between two groups was observed in dietary intakes and physical activity at the baseline and during intervention (Table 2).

**Table 1:** Baseline characteristics of patients with type 2 diabetes

Variables	Intervention (n = 35)	Control (n = 36)	*P*^*a*^
Age, y	50.1 ± 7.3	50.0 ± 7.6	0.96
Diabetes duration, y	9.40 ± 7.0	8.11 ± 6.9	0.44
Gender			0.89
Male, n (%)	20 (57.1)	20 (55.6)	
Female, n (%)	15 (42.9)	16 (44.4)	
Medications			
Metformin, n (%)	30 (85.7)	31 (86.1)	0.96
Glibenclamide, n (%)	11 (31.4)	16 (44.4)	0.25
Statins, n (%)	3 (8.6)	4 (11.1)	0.70
Blood pressure lowering drugs, n (%)	6 (17.2)	5 (13.9)	0.72
Height, cm	164.94 ± 7.2	162.08 ± 11.29	0.20
Weight, kg	73.69 ± 8.2	72.71 ± 10.5	0.66
BMI	27.10 ± 2.6	27.66 ± 2.7	0.39
WC, cm	91.75 ± 7.4	92.58 ± 8.5	0.66
HDL-c, mg/dL	42.49 ± 7.9	45.83 ± 9.0	0.1
TG, mg/dL	188.34 ± 81.8	159.89 ± 63.1	0.1
TC, mg/dL	167.74 ± 33.8	184.03 ± 43.3	0.08
LDL-c, mg/dL	95.86 ± 34.2	105.13 ± 48.7	0.36
LAP	65.62 ± 36.5	55.74 ± 29.4	0.21
VAI	3.29 ± 1.9	2.48 ± 1.2	0.04
CRI-I	4.09 ± 1.0	4.17 ± 1.1	0.78
CRI-II	2.35 ± 0.9	2.42 ± 1.2	0.79
AC	3.09 ± 1.0	3.17 ± 1.1	0.78

Data are expressed as mean ± SD for continuous variables, and as number (percentage) for categorical variables

^a^Differences between the control and intervention groups were evaluated using the independent t-test for continuous variables and chi‐square test for categorical variables

BMI: body mass index; WC: waist circumference; HDL-c: high density lipoprotein-cholesterol; TG: triglyceride; TC: total cholesterol; LDL-c: low density lipoprotein-cholesterol; LAP: lipid accumulation product; VAI: visceral adiposity index; CRI: Castelli risk index; AC: atherogenic coefficient

**Table 2:** Dietary intakes and physical activity in patients with type 2 diabetes

Variables	Intervention (n = 35)	Control (n = 36)	*P*^b^
Energy intake, kcal/d			
Baseline	1612.87 ± 587.87	1708.79 ± 515.39	0.47
After intervention	1544.71 ± 597.37	1674.16 ± 597.07	0.26
* P*^a^	0.45	0.55	
Carbohydrates, %			
Baseline	59.76 ± 12.71	59.82 ± 9.96	0.70
After intervention	60.66 ± 11.20	59.90 ± 8.76	0.48
* P*^a^	0.43	0.88	
Proteins, %			
Baseline	15.05 ± 4.65	15.48 ± 3.48	0.97
After intervention	15.20 ± 5.17	15.84 ± 4.02	0.56
* P*^a^	0.47	0.56	
Fats, %			
Baseline	25.19 ± 14.55	24.70 ± 10.42	0.81
After intervention	24.14 ± 11.02	24.26 ± 9.63	0.63
* P*^a^	0.58	0.77	
Physical activity, MET‐h/d			
Baseline	35.61 ± 5.22	37.54 ± 7.82	0.24
After intervention	36.33 ± 5.70	36.99 ± 5.87	0.62
* P*^a^	0.14	0.31	

Values were presented as mean ± standard deviation (SD)

*P*^a^: resulted from comparisons within groups by paired t-test

*P*^b^: resulted from comparisons between two groups by independent t-test

### Outcomes

The crude analyses did not show any significant change in LAP (P = 0.40), VAI (P = 0.30), CRI-I (P = 0.39), CRI-II (P = 0.34) and AC (P = 0.39) following the intervention (Table 3).

**Table 3:** Effect of resveratrol supplementation on indices in patients with type 2 diabetes

Indices	Intervention			Control			*P*^a^	*P*^b^	*P*^c^	*P*^d^
	Male (n = 20)	Female (n = 15)	Total (n = 35)	Male (n = 20)	Female (n = 16)	Total (n = 36)				
LAP										
Baseline	53.37 ± 24.4	83.13 ± 44.1	65.62 ± 36.5	50.74 ± 22.0	61.68 ± 36.1	55.74 ± 29.4	0.72	0.15	0.21	
After intervention	50.83 ± 25.3	80.76 ± 40.3	63.16 ± 35.1	50.08 ± 21.9	65.60 ± 41.4	57.17 ± 32.7	0.92	0.32	0.46	
* P*	0.39	0.79	0.54	0.81	0.36	0.55				
Mean change of LAP	− 2.54 ± 12.2	− 2.37 ± 34.0	− 2.46 ± 23.3	− 0.65 ± 12.0	3.92 ± 16.8	1.43 ± 14.3	0.63	0.51	0.40	0.43
VAI										
Baseline	2.36 ± 1.1	4.62 ± 2.0	3.29 ± 1.9	2.08 ± 0.9	2.96 ± 1.4	2.48 ± 1.2	0.40	0.01	0.04	
After intervention	2.15 ± 1.1	4.32 ± 1.8	3.04 ± 1.8	1.99 ± 0.8	3.01 ± 1.5	2.46 ± 1.2	0.62	0.04	0.12	
* P*	0.01	0.52	0.20	0.39	0.77	0.82				
Mean change of VAI	− 0.21 ± 0.3	− 0.30 ± 1.7	− 0.25 ± 1.1	− 0.09 ± 0.4	0.05 ± 0.7	− 0.02 ± 0.6	0.39	0.45	0.30	0.47
CRI-I										
Baseline	3.89 ± 1.0	4.39 ± 0.9	4.09 ± 1.0	3.92 ± 1.0	4.45 ± 1.2	4.17 ± 1.1	0.92	0.86	0.78	
After intervention	3.64 ± 1.0	4.12 ± 1.1	3.84 ± 1.0	3.87 ± 1.0	4.33 ± 1.2	4.08 ± 1.1	0.49	0.65	0.38	
* P*	0.31	0.29	0.14	0.68	0.36	0.35				
Mean change of CRI-I	− 0.25 ± 1.0	− 0.27 ± 0.8	− 0.25 ± 0.9	− 0.05 ± 0.5	− 0.12 ± 0.5	− 0.09 ± 0.5	0.49	0.62	0.39	0.79
CRI-II										
Baseline	2.32 ± 0.8	2.40 ± 0.9	2.35 ± 0.9	2.12 ± 0.9	2.78 ± 1.4	2.42 ± 1.2	0.50	0.41	0.79	
After intervention	2.07 ± 0.8	2.20 ± 1.3	2.12 ± 1.0	2.07 ± 1.0	2.69 ± 1.2	2.36 ± 1.1	0.98	0.31	0.40	
* P*	0.20	0.34	0.10	0.74	0.58	0.53				
Mean change of CRI-II	− 0.25 ± 0.8	− 0.20 ± 0.7	− 0.23 ± 0.7	− 0.05 ± 0.6	− 0.09 ± 0.6	− 0.06 ± 0.6	0.38	0.69	0.34	0.38
AC										
Baseline	2.89 ± 1.0	3.39 ± 0.9	3.09 ± 1.0	2.92 ± 1.0	3.45 ± 1.2	3.17 ± 1.1	0.92	0.86	0.78	
After intervention	2.64 ± 1.0	3.12 ± 1.1	2.84 ± 1.0	2.87 ± 1.0	3.33 ± 1.2	3.08 ± 1.1	0.49	0.65	0.38	
* P*	0.31	0.29	0.14	0.68	0.36	0.35				
Mean change of AC	− 0.25 ± 1.0	− 0.27 ± 0.8	− 0.25 ± 0.9	− 0.05 ± 0.5	− 0.12 ± 0.5	− 0.09 ± 0.5	0.49	0.62	0.39	0.79

Values of total CRI-I, CRI-II and AC were presented as mean ± standard deviation (SD), while for LAP and VAI were presented as median and quartile range

LAP: lipid accumulation product; VAI: visceral adiposity index; CRI: Castelli risk index; AC: atherogenic coefficient

*P*: resulted from comparisons within groups by paired t-test

*P*^a^: resulted from comparisons between two groups (males) by independent t-test

*P*^b^: resulted from comparisons between two groups (females) by independent t-test

*P*^c^: resulted from comparisons between two groups (total) by independent t-test

*P*^d^: resulted from comparisons mean changes of variables between two groups after adjusting for medications, diabetes duration, energy intake and physical activity by univariate analysis of covariance (ANCOVA)

After adjusting for confounding variables (medications, diabetes duration, energy intake and physical activity), no significant difference was also revealed in mean changes of LAP (− 2.46 ± 23.3 vs. 1.43 ± 14.3; P = 0.43), VAI (− 0.25 ± 1.1 vs. − 0.02 ± 0.6; P = 0.47), CRI-I (− 0.25 ± 0.9 vs. − 0.09 ± 0.5; P = 0.79), CRI-II (− 0.23 ± 0.7 vs. − 0.06 ± 0.6; P = 0.38) and AC (− 0.25 ± 0.9 vs. − 0.09 ± 0.5; P = 0.79) between groups (Table 3). In addition, a split-sample analysis by gender was performed, and results of all comparisons remained non-significant (Table 3).

## Discussion

The present study found no significant improvement in hepatic and cardiovascular indices following an 8-week 1000 mg/d resveratrol supplementation in patients with T2DM.

It has been confirmed that LAP and VAI can accurately estimate the severity of visceral adiposity and hepatic steatosis [9, 10]. The equations of these validated tools are based on the anthropometric variables and lipid profile [9, 10]. The study of Timmers et al. [40] was the only clinical trial that investigated the effect of resveratrol supplementation on hepatic steatosis among patients with T2DM, and reported that intrahepatic lipid content did not change after resveratrol supplementation (150 mg/d) for 30 days. Some studies have evaluated the effect of resveratrol supplementation in patients with NAFLD. The study of Chachay et al. [31] reported no significant effect of resveratrol supplementation for 8 weeks on hepatic steatosis in patients with NAFLD. However, the study of Faghihzadeh et al. [32], found that 500 mg/d resveratrol supplementation for 12 weeks could reduce the grade of hepatic steatosis in patients with NAFLD. In addition, another study demonstrated a beneficial effect of resveratrol supplementation for 3 months on features of NAFLD [22]. The inconsistence results may be partly related to the wide variation in intervention duration. Clinical trials with longer intervention durations reported the beneficial effect of resveratrol supplementation on hepatic steatosis [22, 32]. Based on the experimental evidence, resveratrol can improve hepatic steatosis through regulating inflammatory pathways, increasing antioxidant capacity and insulin sensitivity, decreasing lipogenic gene expression, de novo lipogenesis and intracellular lipids in the liver, upregulating carnitine/palmitoyl transferase 1 as well as Acyl-CoA oxidase, and subsequent increasing fatty acid (FA) oxidation [25–27, 41].

Resveratrol supplementation could not improve cardiovascular indices such as CRI-I, CRI-II and AC in the present study. These indices are developed based on lipid profile [15, 16]. Some investigations have examined the effect of resveratrol supplementation on cardiovascular indices. The study of Farzin et al. [42] found no significant effect of 12-week resveratrol supplementation (600 mg/d) on CRI-II and AIP in patients with NAFLD. However, the study of Hoseini et al. [30] reported that 500 mg/d resveratrol supplementation can improve TC/HDL-c ratio in patients with T2DM and coronary heart disease. The study of Bo et al. [43] found that resveratrol supplementation has no lipid-modifying effect in patients with T2DM. Another study demonstrated that resveratrol supplementation for 2 months leads to a significant reduction of TC and TG in subjects with dyslipidemia; however, resveratrol had no effect on HDL-C and LDL-c [44]. Consistent with our findings, a recent meta-analysis of RCTs concluded that resveratrol has no impact on dyslipidemia [45]. However, as an important limitation, most of the previous studies did not measure the level of resveratrol in the blood or its metabolites in the urine to assess the bioavailability of the resveratrol. Therefore, the findings must be interpreted with more caution. The experimental investigations suggested that resveratrol can exert its beneficial effects on cardiovascular risk factors by activating adenosine monophosphate kinase (MAPK), increasing fatty acids oxidation, downregulating lipogenic genes, regulating glucose homeostasis, activating the Akt pathway, stimulating intracellular glucose transport, and increasing insulin sensitivity [18, 46–48]. Previously, we reported the effect of resveratrol supplementation on PPARα, p16, p53, p21 gene expressions, and cluster of differentiation 163 (CD163)/ TNF-like weak inducer of apoptosis (TWEAK) ratio in serum [49]. In addition, we reported the findings of anthropometric parameters, lipid profile, atherogenic index of plasma, serum levels of asymmetric de-methyl-arginine and paraoxonase 1 activity [20, 29]. To follow the ethical principals in research, we declare that we used the same data for the present article. To proper understanding and interpretation, Fig. 1 and some important information (baseline characteristics, dietary intakes and physical activity) of our previous articles (20, 29) were added to the present article.

To the best of our knowledge, this is the first study that evaluated the effect of resveratrol supplementation on non-invasive and simple indices such as LAP and VAI in patients with T2DM. In addition, we used micronized trans‐resveratrol, which has higher bioavailability compared to the normal form of resveratrol. However, we conducted a short-term intervention, which is an important limitation of our study. In addition, indices of the present article are the secondary outcomes of our original study, and as mentioned, the optimal sample size was calculated according to a primary outcome. Moreover, we did not measure the level of resveratrol in the blood or its metabolites in the urine to assess the bioavailability of the resveratrol.

In conclusion, 1000 mg/d resveratrol supplementation could not improve hepatic steatosis indices and had no impact on cardiovascular indices; however, it is not mean that resveratrol has no beneficial effect on liver or cardiovascular health. In general, demonstrating the real effect of nutraceuticals on health problems is difficult, especially if the participants are being treated with various medications for their health problems. Moreover, based on the means of lipid profile, most participants in the present study had normal values for serum lipid profile that can complicate the resveratrol's ability to demonstrate efficacy. It is recommended to conduct clinical trials among individuals with higher lipid profile values to reach a definitive conclusion. Furthermore, taking all medications should be monitored and controlled in future clinical trials.

## Data Availability

The data that support the findings of this study are available from the corresponding author, Hassan Mozaffari-Khosravi, upon reasonable request. The data are not publicly available due to their containing information that could compromise the privacy of research participants.

## Abbreviations

AC: Atherogenic coefficient
ANCOVA: Analysis of covariance
CRI: Castelli risk index
CVD: Cardiovascular disease
HbA1c: Glycated haemoglobin
HDL-c: High-density lipoprotein cholesterol
LAP: Lipid accumulation product
LDL-c: Low-density lipoprotein cholesterol
METs: Metabolic equivalents
NAFLD: Non-alcoholic fatty liver disease
PPARα: Peroxisome proliferator-activated receptor alpha
PBMCs: Peripheral blood mononuclear cells
SPSS: Statistical package for social science
T2DM: Type 2 diabetes mellitus
TC: Total cholesterol
TG: Triglyceride
VAI: Visceral adiposity index
